# Life-threatening pediatric retropharyngeal abscess: A case report on delayed complication of endoscopic fishbone removal in an infant

**DOI:** 10.1097/MD.0000000000043664

**Published:** 2025-09-19

**Authors:** Chenyang Xu, Xiaowen Liu, Yufen Guo, Suyang Wang

**Affiliations:** a Department of Otolaryngology-Head and Neck Surgery, Maternal and Child Health Hospital of Gansu Province (Gansu Provincial Central Hospital), Lanzhou, China; b Lanzhou University Second Clinical Medical School, Lanzhou, China; c Department of Otolaryngology-Head and Neck Surgery, Lanzhou University Second Hospital, Lanzhou, China.

**Keywords:** delayed diagnosis, fish bone ingestion, pediatric airway obstruction, postoperative complication, recurrent croup, retropharyngeal abscess

## Abstract

**Rationale:**

Retropharyngeal abscesses (RPAs) following fish bone ingestion are extremely rare in infants under 2 years, with limited cases reported worldwide. Given the anatomical vulnerability and diagnostic challenges faced by infants, this gap in knowledge is critical. No previous reports have described life-threatening delayed RPA caused by endoscopic pharyngeal injury without a retained foreign body, which is a critical omission in the postoperative safety protocols. Our study details a life-threatening delayed RPA caused by posterior pharyngeal wall injury after endoscopic removal of a fish bone, highlights the critical role of postoperative imaging, which is frequently overlooked in pediatric practice, and contributes to the sparse literature on severely delayed RPAs in infants after fish bone ingestion.

**Patient concerns:**

An 18-month-old girl presented with 6 months of recurrent hoarseness, stridor, and progressive respiratory distress.

**Diagnoses:**

The diagnostic evaluations included laboratory studies and imaging examinations, and imaging revealed a large RPA causing significant tracheal compression and pneumonia.

**Interventions:**

Emergency management included intubation, mechanical ventilation, broad-spectrum antibiotics, and surgical drainage.

**Outcomes:**

Surgical drainage and targeted antibiotic therapy resolved inflammation.

**Lessons:**

This novel case of delayed RPA after endoscopic foreign-body removal emphasizes the need for postoperative imaging, urgent surgical intervention, and enhanced caregiver education to prevent infant complications.

## 1. Introduction

Acute upper airway obstruction in young children poses a critical diagnostic challenge when mimicking common viral croup. Retropharyngeal abscesses (RPAs), although rare in children under the age of 2, are life-threatening emergencies that require immediate recognition and intervention.^[1]^ Although foreign body ingestion is a known etiology, delayed fishbone-induced RPAs has not been reported in infants. Existing literature predominantly focuses on acute complications, with limited studies on subacute or chronic sequelae in children.^[2,3]^ This is the first report of life-threatening delayed RPA occurring months after endoscopic fishbone extraction, highlighting a critical gap in the postoperative management. We describe an 18-month-old child with recurrent airway symptoms, initially misdiagnosed with recurrent croup, and ultimately confirmed RPA secondary to esophageal fishbone ingestion 6 months earlier. Despite standard croup therapy, patient deterioration underscores the limitations of clinical assessment alone for differentiating infectious abscesses from viral laryngotracheitis. This report reinforces the necessity of heightened clinical vigilance in pediatric airway emergencies and advocates for systematic approaches, including the judicious use of advanced imaging and adherence to enhanced postoperative evaluation guidelines, to mitigate diagnostic delays and improve outcomes.^[4]^

## 2. Case report

An 18-month-old girl with no notable medical or familial history presented with a 3-day history of worsening hoarseness, stridor, and progressive respiratory distress, which progressed to severe respiratory compromise within 24 hours. Physical examination revealed marked dyspnea, pharyngeal congestion, pronounced inspiratory retraction (suprasternal, supraclavicular, and intercostal), coarse breath sounds in both lungs, and stridor. Refractory hypoxemia (SpO_2_, 66–90%) and tachycardia (160–180 bpm) necessitate emergency intubation. The initial diagnosis was grade III acute laryngeal obstruction potentially secondary to severe laryngitis (Table 1). Initial arterial blood gas (ABG) analysis showed pH 7.30, PaCO₂ 38 mm Hg, PaO₂ 169 mm Hg, K^+^ 4.7 mmol/L, Na^+^ 135 mmol/L, and glucose 6.9 mmol/L. HCO_3_^−^ 18.7 mmol/L, lactate 1.3 mmol/L, BE −7.1 mmol/L, which confirmed metabolic acidosis. Despite nebulized epinephrine, dexamethasone, and intravenous ceftriaxone, clinical deterioration was observed, with bradycardia (57 bpm), profound hypoxemia (SpO₂ 66%), and perioral cyanosis requiring transfer to the pediatric intensive care unit and mechanical ventilation.

**Table 1:** Preoperative grading criteria for laryngeal obstruction.

Grade	Clinical symptom
Grade I	Inspiratory stridor and dyspnea appear only after the exercise
Grade II	Inspiratory stridor and dyspnea appear at rest and aggravate in exercise. Sleeping and eating is not influenced
Grade III	Obvious inspiratory stridor and dyspnea. Dysphoria. Sleeping and eating are influenced. Three concave signs in suprasternal fossa, supraclavicular fossa and intercostal space
Grade IV	Severe dyspnea, cyanosis, unorientation, coma, respiratory failure

Physical examination revealed fever and soft bilateral anterior cervical swelling upon palpation. A separate firm, smooth-edged 2.5 cm mass was palpated in the left cervical region. Urgent ABG analysis showed pH < 7.0, PaCO₂ 92 mm Hg, PaO₂ 117 mm Hg, K⁺ 6.2 mmol/L, Na⁺ 136 mmol/L, glucose 13.8 mmol/L, lactate 14.1 mmol/L (HCO₃⁻/BE uncalculable). Ventilatory support was initiated (pressure support ventilation + synchronized intermittent mandatory ventilation, volume tidal 60 mL, respiratory rate 30/min, FiO_2_ 40%, and positive end-expiratory pressure 5 cm H_2_O). Following stabilization, SpO₂ was normalized to 100%. Repeat ABG at 1 hour demonstrated significant improvement pH 7.31, PaCO₂ 30 mm Hg, PaO₂ 260 mm Hg, K⁺ 4.5 mmol/L, Na⁺ 136 mmol/L, glucose 13.2 mmol/L, lactate 3.6 mmol/L, HCO₃⁻ 15.1 mmol/L, BE −11.2 mmol/L. This indicated that respiratory acidosis and hyperkalemia completely resolved with hemodynamic stabilization. Although cardiopulmonary function improved substantially, metabolic acidosis persisted.

Laboratory studies revealed progressive leukocytosis (white blood cell 19.89 × 10⁹/L), indicating a worsening infection. Hypothermia with refractory hypotension (blood pressure nadir: 71/45 mm Hg) and impaired perfusion signaled evolving septic shock. Empiric antimicrobial therapy was escalated to vancomycin (0.1 g/dose, intravenous, every 6 hours) and Meropenem (0.38 g/dose, intravenous, every 8 hours) for 6 days to cover methicillin-resistant Staphylococcus aureus, other gram-positive, and gram-negative pathogens.^[5–7]^

**Figure 1.**
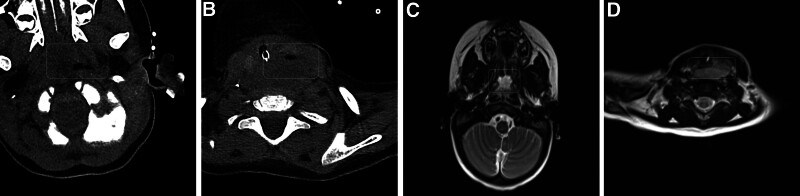
Cervical imaging demonstrating retropharyngeal abscess complications. (A, B) Contrast-enhanced CT: Large cystic lesion compressing the oropharynx, trachea, and left carotid sheath. (C, D) MRI with DWI: Esophageal fistula in the left posterior cervical esophageal wall with an air-fluid level. Maximum compression at the laryngeal-oropharyngeal junction. CT = computed tomography, DWI = diffusion weighted imaging, MRI = magnetic resonance imaging.

Given the diagnostic complexity and clinical deterioration, advanced imaging was performed. Cervical ultrasonography revealed substantial collection of left paraesophageal fluid. Concurrent contrast-enhanced neck computed tomography and magnetic resonance imaging with diffusion-weighted imaging confirmed an esophageal fistula in the left posterior cervical esophageal wall, a large RPA causing significant compression of the oropharynx, trachea, and left carotid sheath, and concomitant pneumonia (Fig. 1). Emergency surgical drainage yielded 50 mL pus. Intraoperative findings revealed diffuse pharyngeal inflammation and a distinct fluctuating mass on the left posterior pharyngeal wall. *Pseudomonas aeruginosa* isolated from pus cultures guided the targeted antibiotic therapy.

**Figure 2.**
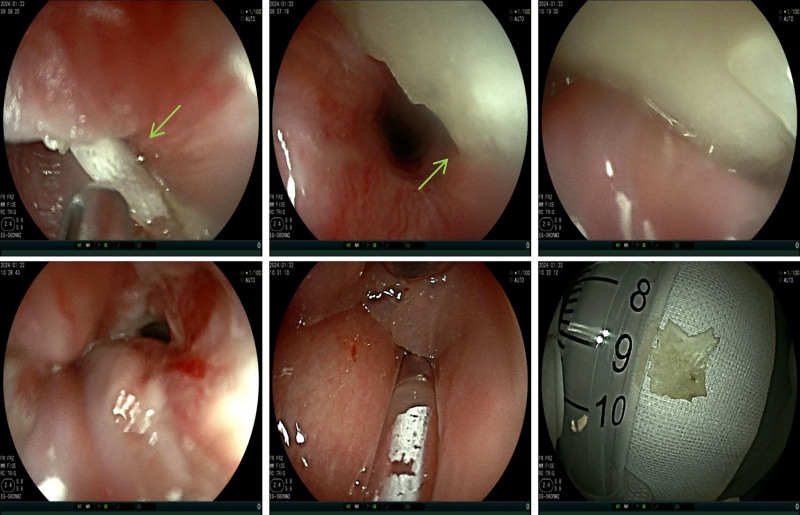
Endoscopic findings during initial fishbone extraction: Impacted fishbone (green arrow) at the entrance of the esophagus with purulent exudate. Granulation tissue and ulceration at the impaction site, distal esophagus, stomach, and duodenum showed normal mucosa. Extracted fishbone (scale reference: 10 mL syringe).

Notably, endoscopic removal of a fish bone from the esophageal entrance was performed 6 months prior, with confirmed extraction (Fig. 2). Postoperative imaging was not performed. Persistent hoarseness and stridor recurred intermittently over 6 months, consistent with recurrent croup despite unresolved symptoms following glucocorticoid therapy. As summarized in Table 2, the delayed diagnosis stemmed primarily from the omission of imaging following the initial foreign body removal attempt and the attribution of persistent symptoms to benign entities. Post-drainage and targeted antibiotics resulted in the complete resolution of the abscess, inflammation, and sepsis. The patient was successfully extubated and recovered completely.

**Table 2:** Timeline of key clinical events.

Timeline	Clinical events
6 mo preadmission	Endoscopic fishbone removal; no postoperative imaging
1 wk post-removal	Recurrent hoarseness and stridor misdiagnosed as croup
Ongoing symptoms	Intermittent fever, respiratory distress; treated as recurrent croup (no improvement)
Day 0 (admission)	Severe hypoxemia (SpO₂ 66%); emergency intubation and mechanical ventilation
24 h post-admission	CT/MRI confirmed retropharyngeal abscess; surgical drainage (50 mL pus evacuated)
1 wk post-surgery	Inflammation resolved; extubation and recovery
Discharge	Caregiver education on hazardous foods; advised follow-up imaging

## 3. Discussion

Pharyngeal and esophageal foreign bodies are common pediatric emergencies, particularly in children under the age of 3 years, and manifest with heterogeneous symptoms spanning the respiratory and gastrointestinal systems.^[8]^ In contrast to blunt objects, sharp or corrosive foreign bodies pose additional risks of mucosal perforation, secondary infection, and abscess formation with potential progression to mediastinitis or vascular injury.^[8–10]^ Although fishbone-induced RPAs are rare in infants, their perforation potential remains significant owing to their sharp, thin, and often fragmented nature.^[1]^

RPAs are frequently misdiagnosed as recurrent croups because of overlapping stridor and respiratory distress.^[2,11]^ Crucially, recurrent croup represents a symptom complex rather than a discrete disease entity, characterized by repeated episodes of barking cough, stridor, and hoarseness, typically occurring more than twice annually.^[12]^ Such recurrences often signify an underlying structural or inflammatory airway abnormality, warranting a thorough investigation into potential etiologies such as congenital subglottic stenosis, laryngomalacia, airway hemangiomas, and gastroesophageal reflux disease.^[13]^ In contrast to the typical viral self-limiting episodes of croup, which are often responsive to glucocorticoids, RPAs present with progressive symptoms that are unresponsive to such therapy and are frequently accompanied by systemic signs of infection (fever, leukocytosis, and elevated inflammatory markers) and potential airway compromise, as exemplified in this case.^[2,14]^ The patient’s 6-month history of recurrent stridor and hoarseness, coupled with a history of fish bone ingestion, was mistakenly attributed to croup, which significantly delayed the diagnosis of the underlying abscess. This diagnostic delay underscores the fact that unrecognized injuries during endoscopy can lead to delayed complications. This case is distinguished from previous reports by the extended latency period, as most reported fishbone-related complications present acutely or subacutely (days to weeks) and not months later.

Cervicothoracic imaging (computed tomography or magnetic resonance imaging) is indispensable for differentiating RPAs from croup or other causes of stridor, and for evaluating the extent of the disease and potential complications, such as mediastinitis or vascular involvement.^[15]^ Anatomically, the retropharyngeal space proximity to the carotid sheath and mediastinal danger space, which leads to the mediastinum, increases the risk of delayed diagnosis.^[4]^ Abscess extension into these spaces can compress neurovascular structures or track inferiorly, potentially leading to catastrophic complications such as carotid artery erosion, jugular vein thrombosis, or mediastinitis.^[16–18]^

Prompt and appropriate interventions, adequate surgical drainage, and culture-guided antibiotic therapy are crucial for preventing life-threatening sepsis and airway obstruction.^[18]^ This case emphasizes the need for meticulous history-taking, particularly in pediatric patients with recurrent respiratory symptoms, and advocates for postoperative imaging following endoscopic removal of sharp foreign bodies, particularly in infants and young children, to exclude residual injuries. NASPGHAN^[8]^ and ESPGHAN^[19]^ typically recommend clinical observation after sharp foreign body removal, without mandatory routine postoperative imaging evaluation. This case demonstrates that infants with persistent unexplained symptoms, who fail to respond to conventional management, warrant thorough investigation. Timely imaging to exclude small perforations, retained fragments, or developing abscesses, even months after the initial event, and long-term surveillance are necessary given the potential for delayed complications.

This report has several constraints. The causative mechanism of pharyngeal perforation, whether iatrogenic or secondary to migrated residual fragments, could not be determined due to the unavailability of endoscopic video records and immediate post-procedural imaging. In the acute deterioration phase, the absence of bronchoscopy prevented us from assessing tracheal mucosal injury. Functional recovery was inadequately characterized by the lack of objective measures and long-term assessments, including video fluoroscopic swallowing study, fiberoptic endoscopic evaluation of swallowing,^[20]^ laryngeal electromyography,^[21]^ and endoscopy. These limitations underscore the need for standardized protocols for managing pediatric foreign-body complications.

## 4. Conclusion

RPAs secondary to ingested sharp foreign bodies are life-threatening pediatric emergencies that require rapid, multidisciplinary intervention. This exceptionally rare case of delayed RPA occurring 6 months after endoscopic fishbone removal highlights 3 critical lessons. Persistent respiratory symptoms in young children with a foreign body history necessitate imaging to exclude abscess formation, regardless of perceived procedural success. Given the anatomical continuity of the retropharyngeal space with the mediastinum and its proximity to neurovascular structures, urgent cross-sectional imaging and surgical drainage are imperative to prevent mediastinitis and sepsis. Finally, prevention remains paramount and depends on meticulous endoscopic examinations and caregiver education to avoid bone-containing foods in young children. The integration of these strategies is essential for mitigating morbidity in pediatric foreign body emergencies.

## Acknowledgments

We thank the patient and her family for their invaluable cooperation and participation and our colleagues for their support.

## Author contributions

**Conceptualization:** Chenyang Xu.

**Data curation:** Chenyang Xu.

**Formal analysis:** Chenyang Xu.

**Funding acquisition:** Xiaowen Liu, Yufen Guo, Suyang Wang.

**Investigation:** Chenyang Xu.

**Methodology:** Chenyang Xu.

**Project administration:** Xiaowen Liu, Suyang Wang.

**Resources:** Suyang Wang.

**Supervision:** Xiaowen Liu.

**Visualization:** Chenyang Xu.

**Writing – original draft:** Chenyang Xu.

**Writing – review & editing:** Chenyang Xu, Yufen Guo, Suyang Wang.
